# *Plasmodium knowlesi* transmission: integrating quantitative approaches from epidemiology and ecology to understand malaria as a zoonosis

**DOI:** 10.1017/S0031182015001821

**Published:** 2016-01-28

**Authors:** P. M. BROCK, K. M. FORNACE, M. PARMITER, J. COX, C. J. DRAKELEY, H. M. FERGUSON, R. R. KAO

**Affiliations:** 1 Institute of Biodiversity Animal Health and Comparative Medicine, College of Medical Veterinary and Life Sciences, University of Glasgow, Glasgow, UK; 2 London School of Hygiene and Tropical Medicine, London, UK

**Keywords:** malaria, mosquito, vector, zoonosis, *Plasmodium knowlesi*, macaque, infectious disease transmission, mathematical model, *R*_0_, reproduction number

## Abstract

The public health threat posed by zoonotic *Plasmodium knowlesi* appears to be growing: it is increasingly reported across South East Asia, and is the leading cause of malaria in Malaysian Borneo. *Plasmodium knowlesi* threatens progress towards malaria elimination as aspects of its transmission, such as spillover from wildlife reservoirs and reliance on outdoor-biting vectors, may limit the effectiveness of conventional methods of malaria control. The development of new quantitative approaches that address the ecological complexity of *P. knowlesi*, particularly through a focus on its primary reservoir hosts, will be required to control it. Here, we review what is known about *P. knowlesi* transmission, identify key knowledge gaps in the context of current approaches to transmission modelling, and discuss the integration of these approaches with clinical parasitology and geostatistical analysis. We highlight the need to incorporate the influences of fine-scale spatial variation, rapid changes to the landscape, and reservoir population and transmission dynamics. The proposed integrated approach would address the unique challenges posed by malaria as a zoonosis, aid the identification of transmission hotspots, provide insight into the mechanistic links between incidence and land use change and support the design of appropriate interventions.

## INTRODUCTION

*Plasmodium knowlesi*, a malaria parasite of monkeys in South East Asia, was identified as an emergent public health threat in 2004 (Singh *et al*. 2004). Previously, *P. knowlesi* in humans was known only from experimental infections carried out for clinical research on neurosyphilis (Collins, 2012), and what was presumed to be a single anomalous spillover event (Chin *et al*. 1965; Vythilingam, 2012). However, there has been a recent increase in the number of human *P. knowlesi* cases reported, particularly in Malaysia (William *et al*. 2013, 2014). The majority of these have been reported in Sarawak and Sabah, where *P. knowlesi* is now the most common cause of clinical malaria (William *et al*. 2013), but cases have also been widely documented in Peninsula Malaysia (Yusof *et al*. 2014). Although Malaysia has achieved malaria pre-elimination status (World Health Organisation, 2014), there is concern that *P. knowlesi* may hinder further progress towards elimination (William *et al*. 2013).

Outside Malaysia, human *P. knowlesi* cases have been reported from across South East Asia (Cox-Singh and Singh, 2008; Moyes *et al*. 2014; Cramer, 2015). However, as diagnosis by microscopy has been rarely confirmed by molecular techniques in regions other than Malaysian Borneo, the overall human burden of *P. knowlesi* infection may be substantially underestimated (Moyes *et al*. 2014). Although artemisinin-based combination therapies clear *P. knowlesi* parasites effectively, a high proportion of patients with *P. knowlesi* develop severe malaria (William *et al*. 2011; Barber *et al*. 2013), and infection can be fatal (Cox-Singh *et al*. 2008; Rajahram *et al*. 2012).

Genetic evidence suggests that *P. knowlesi* derives from an ancestral parasite population that predates the human settlement of South East Asia and, therefore, that *P. knowlesi* may have spilled over into humans prior to 2004, but was misidentified as *Plasmodium malariae* (Lee *et al*. 2009*a*, 2011). Even considering changes in diagnostics and surveillance, notifications of *P. knowlesi* cases in Malaysian Borneo have increased since 2004, which may reflect an increase in incidence (William *et al*. 2014). Land use change has been suggested as a key driver of this increase (Cox-Singh and Singh, 2008), and a recent analysis provides the first evidence for this, indicating that forest cover and loss are associated with *P. knowlesi* incidence (Fornace *et al.* 2016). It has also been suggested that *P. knowlesi* incidence has increased due to a loss of cross-protective immunity following successful control of *Plasmodium vivax* and *Plasmodium falciparum* in Malaysian Borneo (Cox-Singh, 2012; Barber *et al*. 2013), an effect that could act in combination with the influence of land use change, but for which there is little evidence at present (William *et al*. 2013).

Land use change has been hypothesized to influence malaria incidence through a variety of mechanisms (Guerra *et al*. 2006). In the case of *P. knowlesi*, this could involve effects on humans, vectors, primary reservoir hosts – Long-tailed (*Macaca fascicularis*) and Pig-tailed macaques (*Macaca nemestrina*) – and their interactions. For example, changes to human behaviour associated with land-use change could bring humans into closer contact with forest-associated anopheline vectors (Collins, 2012; Barber *et al*. 2013). Migration to new areas may increase contact between humans and the *P. knowlesi* forest transmission cycle, giving rise to ‘frontier’ malaria (De Castro *et al*. 2006). A further possibility is that land-use change influences the transmission dynamics of *P. knowlesi* in a more fundamental way, through effects on the life-history, population dynamics or behaviour of the reservoir host, vector or both. For example, deforestation may cause macaques to crowd into remaining forest patches (Chapman *et al*. 2005; Fooden, 1995), spend more time on the ground (Singh *et al*. 2001) and alter their ranging behaviour and microhabitat use (Riley, 2008). Macaques may also seek out human settlements to raid crops or forage around houses (Chapman and Peres, 2001; Hambali *et al*. 2012), bringing them closer to people. The abundance and community composition of vectors is also associated with land use (Chang *et al*. 1997; Overgaard *et al*. 2003; Petney *et al*. 2009; Brant, 2011), and vectors may reproduce, survive and bite at different rates in anthropogenically altered habitats (Chang *et al*. 1997; Patz *et al*. 2000; De Castro *et al*. 2006).

The large age range of *P. knowlesi* patients and the existence of family clusters of cases suggest that transmission may occur peri-domestically (Barber *et al*. 2012). Human-to-human transmission of *P. knowlesi* has been demonstrated experimentally (Chin *et al*. 1968), and *P. knowlesi* gametocytes are formed during human infections (Lee *et al*. 2009*b*; Van Hellemond *et al*. 2009). However, there is – so far – no evidence from the field of natural human-vector-human transmission (Kantele and Jokiranta, 2011; Vythilingam, 2012; Grigg *et al*. 2014*a*; Ramasamy, 2014). The higher number of *P. knowlesi* genotypes found in macaques compared with humans is consistent with this, as it suggests a higher transmission rate in macaques relative to humans, as does the lack of clustering of *P. knowlesi* genotypes in either host species (Lee *et al*. 2011; Divis *et al*. 2015; Millar and Cox-Singh, 2015).

Here we discuss quantitative approaches to understanding *P. knowlesi* transmission, with an emphasis on mathematical models of transmission, and how they relate to empirical–statistical approaches such as those used to map infectious disease risk. We identify knowledge gaps, and discuss how efforts towards the integration of clinical parasitology, geostatistical analysis and the mathematical modelling of transmission dynamics may yield insight into the epidemiology of *P. knowlesi*. The goal of such efforts should be to identify the determinants of *P. knowlesi* infection risk in an ecological context, so that they can be effectively mitigated to reduce the associated burden of disease.

## QUANTITATIVE APPROACHES TO UNDERSTANDING *P. KNOWLESI* TRANSMISSION

Mathematical models have contributed fruitfully to the understanding of infectious disease transmission (Heesterbeek and Roberts, 2015; Heesterbeek *et al*. 2015), and much of the research effort on controlling mosquito-borne disease during the last century has focused on the development of transmission models (Mandal *et al*. 2011; Smith *et al*. 2012; Reiner *et al*. 2013; Wallace *et al*. 2014). Contemporary malaria models are increasingly geared towards including the geographical, ecological and epidemiological heterogeneities that influence transmission. These models can address questions about the relative merits of interventions (e.g., Griffin *et al*. 2010), and have played an important role in reducing mortality and morbidity around the world (World Health Organisation, 2014). However, a recent review (Reiner *et al*. 2013) revealed that most mosquito-borne disease models are strikingly similar as they share central assumptions made by the earliest malaria models, collectively referred to as Ross–MacDonald. These drove the development of pathogen transmission theory, the formulation of metrics such as *R*_0_ and vectorial capacity, and allowed for prediction of the effectiveness of mosquito-borne disease control (MacDonald *et al*. 1968; Smith *et al*. 2012). However, the assumptions of Ross–MacDonald models make them less applicable to the transmission of diseases with complex ecologies such as *P. knowlesi*.

The simplest way to include a wildlife reservoir in a Ross–MacDonald model is by adding a non-human host compartment, allowing for host-specific transmission rates, and this was the approach taken by an early transmission model of *P. knowlesi* (Yakob *et al*. 2010). There is growing general interest in understanding how different host species contribute to the persistence of pathogens in multi-host systems, so that control can be appropriately targeted (Haydon *et al*. 2002; Fenton and Pedersen, 2005; Streicker *et al*. 2013; Viana *et al*. 2014). To describe *P. knowlesi* transmission in the context of this trend in disease ecology and test the broad plausibility of natural human–vector–human transmission, we developed a scenario exploration tool for this review using a model similar to Yakob *et al*. 2010. Following Imai *et al*. (2014), we assessed *P. knowlesi* transmission scenarios with variable levels of human–vector–human transmission according to their human and macaque equilibrium prevalences (Box 1). The results suggest that natural human–vector–human transmission of *P. knowlesi* is plausible, but that it is likely to be rare, which is consistent with previous work (Imai *et al*. 2014).

Box 1. Modelling transmission scenarios

To describe *P. knowlesi* transmission in terms of disease ecology, a Ross–MacDonald-type model was used to compare the plausibility of transmission scenarios with variable rates of human–vector–human transmission. Following Yakob *et al*. (2010) and Imai *et al*. (2014), a differential equation model tracked the proportions of infected macaques, vectors and humans through time. The daily vector mortality rate (*g* = 0·15), the extrinsic incubation period (*T* = 10 days), and the daily mammalian recovery rate (*r* = 0·07) were fixed at values considered typical for human malarias for lack of suitable data on *P. knowlesi*. To take into account variation in remaining variables and parameters, 100 000 sets of values were sampled from the following ranges using Latin hypercube sampling: number of humans, *H*, (2000–200 000); number of macaques, *M*, (2000–200 000); number of vectors, *V*, (2000–200 000); transmission coefficients: vector–human, *C*_*vh*_, (0–1), vector–macaque, *C*_*vm*_, (0–1), human–vector, *C*_*hv*_, (0–1), macaque–vector, *C*_*mv*_, (0–1); vector-biting preference for humans *vs* macaques, *p*, (0–1); biting rate (per mosquito per day), *b*, (0–7).

Models were initiated with infection prevalences of 1, 0 and 0% in the vector, macaque and human populations, respectively, and run to equilibrium for each scenario. Parameter sets were considered to be plausible if they generated equilibrium prevalences of 0·5–5% in humans (based on empirical data from cross-sectional surveys and active case detection in Kudat, Sabah [Fornace *et al*. 2015]) and 50–90% in macaques. The macaque prevalence range was intentionally broad, given the limited and variable nature of available estimates (Vythilingam *et al*. 2008; Jeslyn *et al*. 2011; Lee *et al*. 2011). In a departure from previous *P. knowlesi* models, we calculated the average number of secondary human infections caused by a single macaque infection, *R*^MH^, and by a single human infection, *R*^HH^, for each scenario:

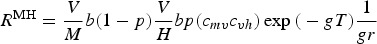


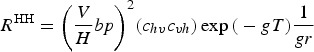

*R*_0_ for each scenario was calculated as the dominant eigenvalue of the next generation matrix (NGM; Diekmann *et al*. 2010; Brooks-Pollock and Wood, 2015):

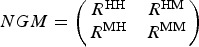

where

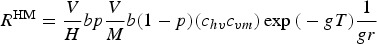


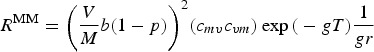

**Fig. 1.**
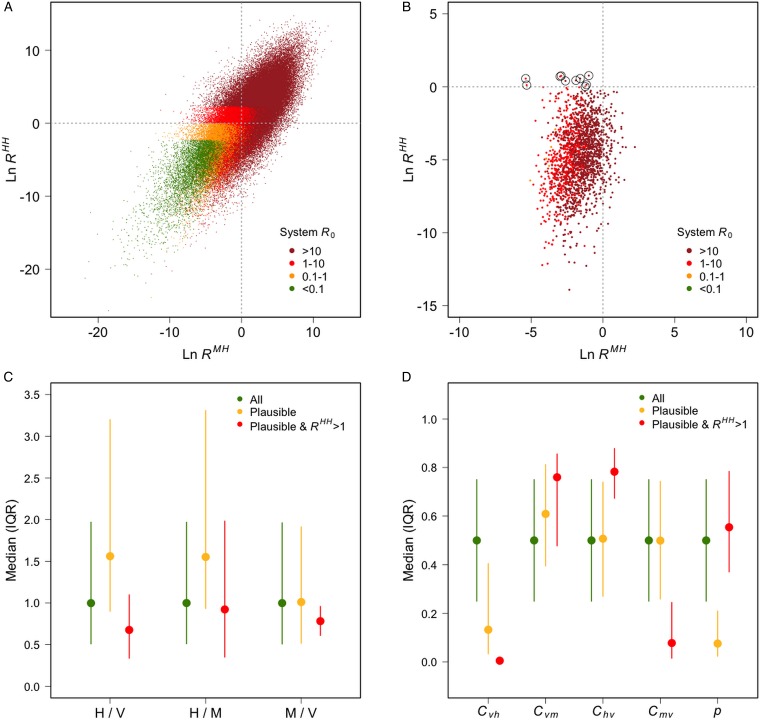
(A) The average number of secondary human infections caused by a single macaque case (*x*-axis) and by a single human case (*y*-axis), and system *R*_0_ (colours), for each scenario; (B) the same information plotted only for scenarios that generated prevalences deemed plausible (humans: 0·5–5%; macaques: 50–90%), scenarios in which *R*^HH^ was >1 are circled; (C) the medians and interquartile ranges of the ratios of humans to vectors, humans to macaques and macaques to vectors for all scenarios, plausible scenarios, and plausible scenarios in which *R*^HH^ >1; (D) the median and interquartile ranges of the four transmission coefficients: *C*_*vh*_ (vector–human), *C*_*vm*_ (vector–macaque), *C*_*hv*_ (human–vector) *C*_*mv*_ (macaque–vector); and the vector-biting preference for humans *vs* macaques (*p*).

The observed asymmetrical distribution of system *R*_0_ (Fig. 1a) is as expected, given that *R*^HH^ is an element of the primary, while *R*^MH^ is an element of the secondary, diagonal of the NGM. 1837 of 100 000 scenarios generated plausible human and macaque prevalences. *R*^HH^ was greater than one in ten of these plausible scenarios (Fig. 1b), *R*^MM^ was less than one in eight of these plausible scenarios, and both of these conditions were met in only two plausible scenarios. These results suggest that human infections are more likely to result from zoonotic transmission, but that natural human–vector–human transmission cannot be ruled out, and that humans are unlikely to play a role in parasite maintenance. This is consistent with previous work (Imai *et al*. 2014) and builds on it by estimating the relative contributions of each host species to transmission.

Plausible scenarios were characterised by high numbers of humans, low vector–human transmission, high vector–macaque transmission and low human-biting preference (Fig. 1c, d). The few plausible scenarios in which *R*^HH^ was greater than 1 were characterized by high numbers of vectors, and a combination of low vector–human and macaque–vector transmission with high vector–macaque and human–vector transmission (Fig. 1c, d).

Although designed for demonstration rather than inference, this model shows how key aspects of *P. knowlesi* transmission could be identified and used to inform control and surveillance programmes. For example, high numbers of vectors may increase the risk that sustained human-to-human transmission emerges. In this case, the influence of land use change on vector population dynamics may be an important determinant of human disease risk, and vector control may be an effective intervention option.

### Spatial distribution of hosts and vectors

Although useful for illustrative scenario exploration, the simplifying assumptions of models such as that used in Box 1 mean they are unable to incorporate fundamental aspects of *P. knowlesi* transmission. For example, they assume that host populations are well mixed, and that mosquitoes feed randomly on vertebrate hosts (Reiner *et al*. 2013; Perkins *et al*. 2013). However, human populations are unlikely to be homogenously mixed with either the forest-associated vectors of *P. knowlesi*, or macaque reservoir hosts (Jiram *et al*. 2012; Wong *et al.* 2015). The omission of spatial variation from models of systems in which it is has an important influence on transmission can bias estimation of epidemiological parameters (Meyers *et al*. 2005; Riley, 2007; Riley *et al*. 2015). Spatial variation may be particularly important to include in models of zoonoses (Alexander *et al*. 2012, 2015) and vector-borne disease (Perkins *et al*. 2013), as their transmission involves multiple species with distinct ecologies. Therefore, ignoring heterogeneity in the spatial distribution of hosts, vectors and vector-biting patterns could introduce problematic bias into analysis and prediction of *P. knowlesi* transmission.

Efforts to incorporate spatial heterogeneity into understanding of *P. knowlesi* transmission have drawn on research into dengue and yellow fever, which like *P. knowlesi* are vector-borne and have forest transmission cycles (Gubler, 2004; Vasilakis *et al*. 2011). Imai *et al*. (2014) modelled transmission across three zones (forest, farm and village), in which the relative abundance of macaque and human hosts was varied. Macaques were assumed to range across the forest and farm zones, humans were assumed to be present in the farm and village zones, and a separate vector population was modelled in each zone. The model predicted that humans were at greatest risk of *P. knowlesi* infection when macaques were present in sufficient numbers in both the forest and the farm zones, as this allowed for the maintenance of infection in the forest, and its dissemination to humans at farms (Imai *et al*. 2014). The explicit consideration of the interface between the forest transmission cycle of *P. knowlesi* and the at-risk human population lays valuable groundwork for the identification of the key interactions that determine human infection risk. However, compartmental zonation does not take into account the heterogeneous spatial co-distribution of habitats or land use types in, for example, Malaysian Borneo, where farming practices and settlement proximity to forest are variable. Therefore, to understand *P. knowlesi* transmission dynamics in space, research will need to look beyond a compartmental modelling framework.

One way to capture fine-scale spatial variation in infectious disease risk is using advanced cartographic techniques, such as occurrence mapping and model-based geostatistics (Patil *et al*. 2011; Hay *et al*. 2013; Pigott *et al*. 2015; Dalrymple *et al*. 2015). In contrast to mechanistic transmission models (such as Ross–MacDonald), these methods use empirical models (i.e., statistical models that do not make assumptions about mechanism) to quantify the association between disease occurrence or transmission metrics and spatially dependent explanatory variables. These models vary in form and can take into account the auto-correlated nature of spatial data, and other sources of bias such as unevenly distributed variation (Pullan *et al*. 2012; Hay *et al*. 2013; Wardrop *et al*. 2014). Such mapping techniques have been widely used to predict infectious disease risk by estimating transmission metrics (e.g., Clements *et al*. 2006; Hay *et al*. 2009; Vounatsou *et al*. 2009; Gething *et al*. 2011; Lau *et al*. 2012; Raso *et al*. 2012; Wardrop *et al*. 2013; Grimes and Templeton, 2015; Lai *et al*. 2015) and occurrence (e.g., Bhatt *et al*. 2013; Cano *et al*. 2014; Gilbert *et al*. 2014; Pigott *et al*. 2014; Messina *et al*. 2015; Mylne *et al*. 2015) in space, and have been used to guide disease control policy and practice (e.g., Diggle *et al*. 2007; Magalhães *et al*. 2011). However, these techniques are not problem-free as they make implicit assumptions about the systems to which they are fitted (Wardrop *et al*. 2014). The most relevant of these assumptions for *P. knowlesi* is that the disease being mapped is assumed to be in equilibrium with its environment. To some extent, this can be dealt with by iteratively re-fitting models as new data become available (e.g., Gething *et al*. 2011; http://www.abraid.ox.ac.uk). For diseases that are not at equilibrium, though, iteratively re-fitting models that do not incorporate a mechanistic description of transmission may not lead to a cumulatively clearer picture of risk (Wood *et al*. 2015). Widespread and rapid land use change of the kind currently occurring in Malaysian Borneo (Langner *et al*. 2007; Bryan *et al*. 2013; Fornace *et al*. 2014) could give rise to this kind of unstable epidemiology. Therefore, insight from both empirical and mathematical modelling approaches (Karesh *et al*. 2012) will be required to understand spatial heterogeneity in human risk of *P. knowlesi* infection.

A constructive way in which quantitative methods from across disciplines may be integrated is through the development of new types of vector-borne disease transmission models. Increasing recognition of the importance of heterogeneity in aspects of vector-borne disease transmission supports an argument for ‘recasting’ standard models by switching their focus from transmission onto host and vector movement, and the ecological and social variation that drive them (Smith *et al*. 2014). Such a shift in emphasis could simultaneously address the problems of the uneven distribution of vector bites amongst vertebrate hosts, non-random mixing of hosts and vectors, and finite host population sizes, which are not taken into account (simultaneously) by current models (Perkins *et al*. 2013). This approach may be particularly applicable to the investigation of zoonotic infections such as *P. knowlesi*: due to constraints on the flexibility of models to incorporate their interface, transmission models of zoonoses have tended to focus either on dynamics in the reservoir or outbreaks in the target population (Lloyd-Smith *et al*. 2009; Lloyd-Smith *et al*. 2014). The re-organization of model mechanics proposed by Smith *et al*. (2014) may allow for the simultaneous estimation of reservoir transmission dynamics, spillover events, and onward transmission in the target population. In addition, it may allow for the incorporation of stochasticity, which is particularly important for systems in which rare events (e.g., spillover) may have high impact (Lloyd-Smith *et al*. 2005; Smith, 2008; King *et al*. 2015). However, although promising, it remains to be seen whether the requisite fine-scale spatial data on *P. knowlesi* host and vector movement will be available, and therefore whether this data-intensive approach will provide further insight into the dynamics of *P. knowlesi* transmission.

### Spatial scales of transmission

Epidemiological and ecological processes that influence zoonotic disease transmission operate over different spatial scales; for example, on average humans move over greater distances than mosquitoes. In addition to variability in the static spatial distribution of hosts and vectors discussed above, variation in their movement is likely to have an important influence on vector-borne disease transmission. This is because it affects contact between individuals, the distances over which parasite is moved, and the spatial scales over which individuals interact with the environment.

Compartmental models of malaria transmission, such as Ross–MacDonald, implicitly assume that mixing between hosts and vectors is homogenous at a defined spatial scale. The results of such models, therefore, are likely to be misleading if related to real world scenarios in which spatial scale assumptions are unjustified; for example, if an area into and out of which hosts migrate is modelled as self-contained. Bias introduced by the spatial scale assumptions of transmission models has been shown to interact with the degree of clustering of hosts in space (Perkins *et al*. 2013), and how it influences the estimation of transmission metrics such as incidence is likely to vary between pathogen–host systems (Mills and Riley, 2014; Riley *et al*. 2015). For vector-borne pathogens and zoonoses, such as *P. knowlesi*, the multiplicity of spatial scales implicated in transmission may be particularly problematic, as they involve multiple species with varied ecologies (Lambin *et al*. 2010; Ben-Ari *et al*. 2011; Johnson *et al*. 2015).

To illustrate the challenge spatial scale presents to understanding transmission, we used data from a case–control study carried out in Kudat, Malaysian Borneo (Grigg *et al*. 2014*a*) to analyse how the strength of association between key environmental variables and human *P. knowlesi* infection status varied with scale (Box 2). The results show that different environmental variables were most strongly related to infection status when summarized over areas of different sizes. The exercise illustrates how spatial scales that characterize transmission may be identified, and suggests that incorporating multiple scales into inferential and predictive analysis of *P. knowlesi* transmission may be beneficial. In addition, this approach could be used for designing disease interventions: the scale at which the density of aquatic breeding sites was most strongly associated with infection status, for example, could inform the deployment of larvicidal control.

Box 2. Spatial scale of environmental risk factors

To illustrate the problem of variable influence on transmission across multiple spatial scales, we used data from a case–control study in Kudat, Malaysian Borneo (Grigg *et al*. 2014*a*) to explore how the strength of association between key environmental features and human *P. knowlesi* infection status changed with spatial scale. Forest cover and loss variables were extracted from classified satellite data (Hansen *et al*. 2013) for neighbourhoods of varying size (radii 2·1–5·5 km) around households where *P. knowlesi* was reported. To compare with case household locations, the same forest variables were extracted from neighbourhoods around points randomly sampled in space (pseudo-absences, Barbet-Massin *et al*. 2012) from inside the catchment area of the case–control study. A threshold of >50% canopy cover was used to define forest, and the four variables calculated were: proportional forest loss (2000–2012), fragmentation of forest lost (perimeter area ratio, 2000–2012), proportional forest cover (2012) and fragmentation of cover (perimeter area ratio, 2012). Each environmental variable was fitted as the only explanatory variable in a generalized additive model of infection probability (case *vs* pseudo-absence) for all neighbourhood sizes (2·1–5·5 km).

**Fig. 2.**
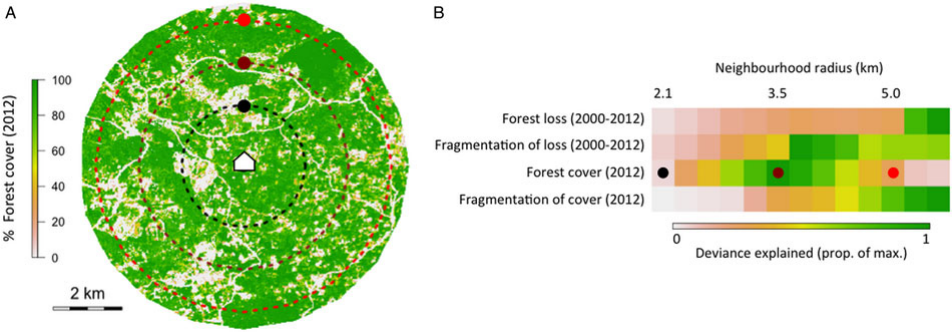
(A) Three example neighbourhood sizes drawn around a case household, showing % forest cover in 2012, and (B) the deviance explained by four example forest variables at 13 neighbourhood sizes in univariate generalized additive models of infection status.

Figure 2a shows neighbourhoods of three example sizes around a household. Figure 2b shows how the strength of the association between environmental variables and infection status changes with spatial scale, and that the scales at which variables have the strongest association with infection status are different. Forest loss was most strongly associated with infection status when summarized over an area with radius 5·5 km, while forest cover was most strongly associated with infection status when summarized over an area with radius of 3·5 km. This analysis is designed to demonstrate the problem of multiple spatial scales for understanding and predicting *P. knowlesi* disease risk. The question of how combinations of relevant spatial scales might be incorporated into analysis and prediction is discussed in the main text, but remains a challenge, and will be the focus of future work.

## CHALLENGES AND OPPORTUNITIES

### Data gaps

Data on human *P. knowlesi* infection are accumulating, particularly in Malaysian Borneo, and understanding of the clinical epidemiology of *P. knowlesi* is improving (Daneshvar *et al*. 2009; Barber *et al*. 2012, 2013; William *et al*. 2013, 2014; Grigg *et al*. 2014*a*, *b*). However, most data are from hospitals and clinics, and there is a shortage of community-based estimates of human infection patterns. Therefore, *P. knowlesi* transmission is only beginning to be understood in the ecological context of spillover from wildlife (Fornace *et al.* 2016). The major challenge facing this effort is the unbiased collection of data on human infections, reservoir hosts and vectors of *P. knowlesi* of sufficient geographical spread for integration into a generalized model of *P. knowlesi* transmission. This review has focusses on the details of the quantitative methods that might be used for this integration, but without the requisite data, the approaches discussed may not be feasible.

Studies of *P. knowlesi* in macaques suggest that prevalence is particularly high in Malaysia and Long-tailed macaques (Vythilingam *et al*. 2008; Lee *et al*. 2011). However, published *P. knowlesi* prevalence estimates in macaques are based on small numbers of individuals and are variable. In addition, there are few data available on macaque population densities, and how these vary across the geographical range of *P. knowlesi,* which would be required to put prevalence estimates into epidemiological context (Fooden, 1995; Moyes *et al*. 2014). Additionally, data on the habitat preferences, movement patterns and social dynamics of macaques will be necessary for predictions about *P. knowlesi* transmission in future scenarios of land use change. At present, the data available are either not sufficiently specific to *P. knowlesi* (e.g., Chapman and Peres, 2001; Singh *et al*. 2001; Chapman *et al*. 2005; Riley, 2008) or too geographically limited to allow for generalization (e.g., Hambali *et al*. 2012). Macaque data would also allow for deeper exploration of the biting preferences of *P. knowlesi* vectors, through better estimates of host availability. If *P. knowlesi* infectious bites fall on humans because macaques are temporarily absent – and other biteable mammals are permanently absent from degraded forest (Civitello *et al*. 2015; Keesing and Ostfeld, 2015; McCallum, 2015) – this could have implications for interventions aimed at lowering human infection risk, particularly if macaque movement patterns are influenced by human movement patterns.

Similar data gaps exist for vectors, but the challenges associated with addressing them are different. The geographical diversity of the region across which *P. knowlesi* has been reported means extrapolation between areas is inadvisable (Ahmed and Cox-Singh, 2015). This is especially true for vector ecology, as the principal *Anopheles* species responsible for *P. knowlesi* transmission varies locally and between regions. The vectors of *P. knowlesi* belong to the *Anopheles leucophyrus* group: *An. hackeri* has been incriminated in Selangor; *An. introlatus* in Hulu Selangor; *An. cracens* in Kuala Lipis; *An. latens* in Sarawak; *An. balabacensis* in Sabah; and *An. dirus* in Vietnam (Vythilingam *et al*. 2006, 2008, 2014; Tan *et al*. 2008; Marchand *et al*. 2011; Jiram *et al*. 2012; Wong *et al*. 2015). Reliable data on vector abundance and behaviour as required to estimate vectorial capacity are limited, and the information that is available is drawn from such different settings that generalization is inadvisable (e.g., Jiram *et al*. 2012; Wong *et al.* 2015). Although there are some data on how the diversity, abundance and life history of *P knowlesi* vectors vary between habitats and respond to anthropogenic disturbance (Chang *et al*. 1997; Petney *et al*. 2009; Brant, 2011; Wong *et al.* 2015), they are not yet detailed or geographically diverse enough to inform predictions of human *P. knowlesi* infection risk under scenarios of future land use change.

In addition, there is little available data on land use to put data on *P. knowlesi* hosts and vectors into geographical context. The collection of land use data of sufficient resolution over sufficiently large areas is challenging given the speed of land-use change in parts of *P. knowlesi*'s range, particularly in Malaysian Borneo (Fornace *et al*. 2014). In addition, the classification of such remotely sensed data poses a problem given that the ecotypes relevant for *P. knowlesi* transmission are – as yet – unknown; the land use categories that best describe variation in macaque behaviour, for example, are likely to be different from those that best describe variation in mosquito life history.

Finally, it is important to consider the added complexity of human behaviour, and how it relates to land use change, and might interplay with other influences on *P. knowlesi* transmission to determine risk.

### Opportunities

Acquisition of detailed data on macaques and vectors across broad geographic scales will require randomized sampling. The same is true of data on humans, which could be achieved by carrying out cross-sectional surveys, and by incorporating *P. knowlesi* screening into existing surveys. This would have numerous benefits such as allowing for the distinction between human and non-human drivers of infection, and taking into account case-reporting bias (Barber *et al*. 2012, 2013; William *et al*. 2013, 2014; Grigg *et al*. 2014*a*). New techniques for *P. knowlesi* diagnosis (Millar and Cox-Singh, 2015) mean randomized sampling would provide data on asymptomatic *P. knowlesi* infection in humans (e.g., Van den Eede *et al*. 2010; Cox-Singh, 2012), which would allow for more accurate estimates of human exposure. It would also allow for estimates of exposure using serology, for which *P. knowlesi*-specific assays are currently being developed. Relevant serological markers maximize the information derived per sample, as antibody responses last longer than infection, and information from markers with different time signatures can be combined (e.g., Helb *et al*. 2015). The ability to control for sampling effort would broaden the scope of genetic studies aimed at tracking parasite diversity and transmission (e.g., Noviyanti *et al*. 2015), and provide the necessary tools for surveillance of natural human–vector–human transmission. Currently, genetic studies on *P. knowlesi* (e.g., Lee *et al*. 2011; Divis *et al*. 2015) use opportunistically collected samples that represent parasite populations to an unknown degree. A randomized sampling design would also allow for the use of more powerful quantitative analysis tools. For example, model-based geostatistical techniques require spatial data on the absence as well as presence of infection, and a broader geographical spread of data would confer greater predictive power. If sufficient environmental variation were incorporated into sampling designs, environmental interpolation would equate to geographical extrapolation, allowing for risk estimation outside of the study area (Matthiopoulos *et al*. 2011; Gilbert *et al*. 2014). Together, the above illustrates the many potential benefits of the incorporation of randomized sampling into data collection efforts on *P. knowlesi*.

Research on zoonotic *P. knowlesi* necessitates the integration of a wider range of disciplines than human malarias. Mathematical models are useful tools for such interdisciplinary integration (McKenzie, 2000), as has been recognized since the earliest days of malaria epidemiology: ‘The aim of mathematical epidemiology is to integrate biological and circumstantial data into one coherent whole’ (MacDonald, 1957). The study of *P. knowlesi* transmission represents an opportunity to link functional ecology with mathematical epidemiology, and combine the strengths of the empirical–statistical approaches largely used by the former with those of the mechanistic models largely used by the latter. The incorporation of functional ecology into descriptions of *P. knowlesi* transmission of the kind being called for in vector-borne disease research in general (Smith *et al*. 2014; Hartemink *et al*. 2014) and malaria research in particular (Ferguson *et al*. 2010; Godfray, 2013; Perkins *et al*. 2013), could inform the design of disease control programs, and provide insight into the processes of zoonotic emergence, the implications of which could be significant beyond *P. knowlesi*.

Functional ecology can be incorporated into predictions of infectious disease risk through analytical focus on the biological resources necessary for pathogens to complete their life cycles (Hartemink *et al*. 2014; Killeen *et al*. 2014). This has the advantages of introducing a spatial dimension often lacking from mathematical models of human malaria transmission and taking into account biological mechanisms often lacking from ecological statistics (Hartemink *et al*. 2014). As applied to *P. knowlesi*, the multiple spatial scales implicated in transmission complicate the incorporation of resource-dependence into spatial predictions of infection risk. However, this multi-scale problem has been identified in other areas of ecological research, including prediction of animal habitat use (Beyer *et al*. 2010), and analytical tools have been developed to deal with it (e.g., Matthiopoulos *et al*. 2011). One such method with potential application to *P. knowlesi* frames animal habitat use in the context of availability (Aarts *et al*. 2013), whereby, for example, the use of forest by macaques on one scale (e.g., a zone of radius of 1 km) is dependent on its availability on another (e.g., a zone of radius 5 km). The application of such an estimation framework to prediction of *P. knowlesi* infection risk would allow both for the variable influence of environmental factors across spatial scales, and for the possibility that environmental factors may have important influences on more than one spatial scale.

Any method seeking insight into *P. knowlesi* transmission needs to consider how fast the landscape is changing in parts of *P. knowlesi*'s range, particularly in Sabah and Malaysian Borneo (Langner *et al*. 2007; Bryan *et al*. 2013; Fornace *et al*. 2014). Another development of ecological theory that could be usefully applied to this problem for predicting *P. knowlesi* infection risk is research into the links between animal habitat use and population dynamics (Morales *et al*. 2010; Matthiopoulos *et al*. 2015). Animals use habitat in different ways depending on their population density and whether it is changing (Matthiopoulos *et al*. 2015). Therefore, if land use change impacts the population densities of mosquitoes and macaques, this may change not only how they are distributed in space, but also the parameters and functional forms that define their distribution in relation to environmental variables. This could have an important influence on human infection risk in an area of rapid land use change such as Malaysian Borneo, and adoption of ecological modelling approaches may be the key to understanding the environmentally dependent epidemiology of zoonoses such as *P. knowlesi*. Such approaches may even be applicable to quantifying human movement and habitat use, and could be explored as a way of plugging sociological studies into an integrated framework for understanding *P. knowlesi* transmission.

### Concluding remarks

Clinical investigation has advanced understanding of *P. knowlesi* epidemiology. To move towards prediction of human infection risk, the next phase of *P. knowlesi* research should focus on its ecology. Aspects of spatial variation in *P. knowlesi* infection risk have begun to be mapped on a broad spatial scale (Moyes *et al*. 2014), and *P. knowlesi* has been identified as a priority disease for future mapping efforts (Pigott *et al*. 2015). Although infectious disease mapping of this kind brings many advantages (Hay *et al*. 2013), given the rapid rates of landscape change in areas where human risk of *P. knowlesi* appears highest, efforts to understand and control *P. knowlesi* transmission will benefit from work on the mechanisms that give rise to spatial patterns of infection risk to complement their empirical–statistical description through mapping. If sufficient appropriate data are available, and spatial heterogeneities can be accounted for, such mechanistic models would confer greater power for extrapolation and prediction, and allow for more effective identification of risk groups and areas, optimization of intervention deployment, and surveillance for natural human–vector–human transmission.
